# Exploring the memory: existing activity-dependent tools to tag and manipulate engram cells

**DOI:** 10.3389/fncel.2023.1279032

**Published:** 2024-01-08

**Authors:** Bo Pang, Xiaoyan Wu, Hailun Chen, Yiwen Yan, Zibo Du, Zihan Yu, Xiai Yang, Wanshan Wang, Kangrong Lu

**Affiliations:** ^1^The Second Clinical Medical College, Southern Medical University, Guangzhou, China; ^2^The First Clinical Medical College, Southern Medical University, Guangzhou, China; ^3^School of Basic Medicine Science, Southern Medical University, Guangzhou, China; ^4^Department of Neurology, Ankang Central Hospital, Ankang, China; ^5^Laboratory Animal Management Center, Southern Medical University, Guangzhou, China; ^6^Guangzhou Southern Medical Laboratory Animal Sci. and Tech. Co., Ltd., Guangzhou, China; ^7^NMPA Key Laboratory for Safety Evaluation of Cosmetics, Southern Medical University, Guangzhou, China

**Keywords:** engram cells, activity-dependent tools, neuronal activity, genetic strategy, memory

## Abstract

The theory of engrams, proposed several years ago, is highly crucial to understanding the progress of memory. Although it significantly contributes to identifying new treatments for cognitive disorders, it is limited by a lack of technology. Several scientists have attempted to validate this theory but failed. With the increasing availability of activity-dependent tools, several researchers have found traces of engram cells. Activity-dependent tools are based on the mechanisms underlying neuronal activity and use a combination of emerging molecular biological and genetic technology. Scientists have used these tools to tag and manipulate engram neurons and identified numerous internal connections between engram neurons and memory. In this review, we provide the background, principles, and selected examples of applications of existing activity-dependent tools. Using a combination of traditional definitions and concepts of engram cells, we discuss the applications and limitations of these tools and propose certain developmental directions to further explore the functions of engram cells.

## 1 Introduction

The term “engrams,” also called memory traces, was first defined by Semon as stimulus-induced enduring physical changes in the brain (Semon, 1921, 1923). Engram cells, or engram neurons, are populations of cells that constitute the critical cellular components of a specific engram. They share the following characteristics: (i) activated by an experience, (ii) physically or chemically modified by the experience, and (iii) required for experience-related memory retrieval (Josselyn and Tonegawa, 2020).

Understanding memory has been a historical and difficult scientific question. A comprehensive study of memory can assist in devising efficient treatment strategies for cognitive disorders, such as Alzheimer's disease (AD; Ludvig, 1997; Scott et al., 2002; Li et al., 2010; Chung et al., 2013; Ramirez et al., 2015; Barron et al., 2017; Záborszky et al., 2018; Nomura, 2020; Zhang et al., 2020; Rao-Ruiz et al., 2021; Buhusi and Buhusi, 2023; Sancho-Balsells et al., 2023; Yan et al., 2023). One of the challenges faced in understanding memory is how to visualize and capture the ever-changing memory traces in the brain at the cellular level. In this regard, the concept of engram cells provided scientists with a meaningful target to study memory (Buhusi and Buhusi, 2023; Dai et al., 2023; Gall et al., 2023; Ghandour and Inokuchi, 2023; Hammack et al., 2023; Jung et al., 2023; Kenna et al., 2023; Lee C. et al., 2023; Mohanta et al., 2023; Nomoto et al., 2023; Okray et al., 2023; Osanai et al., 2023; Park et al., 2023; Rahsepar et al., 2023; Schott, 2023; Terranova et al., 2023; Wilmerding et al., 2023). In the 1950's, certain scientists could not identify engram cells due to a lack of technology (Lashley, 1931, 1950, 1963). A combination of ever-advancing activity-dependent tools and genetic strategies has resulted in significant progress in understanding different memory stages at the engram level (Josselyn et al., 2015; Tonegawa et al., 2015, 2018; Roy et al., 2016; Frankland et al., 2019; Vetere et al., 2019; Josselyn and Tonegawa, 2020; Ryan et al., 2021) and providing different and novel treatment ideas for AD (Roy et al., 2016; Bostanciklioglu, 2020a,b; Poll et al., 2020; Mishra et al., 2022).

To examine the presence and study the physiological mechanisms underlying the functioning of engram cells, scientists created a class of tools known as activity-dependent tools. These are based on the definition of engram cells and the molecular mechanisms contributing to neural activity (Bai and Suzuki, 2020; Colecraft, 2020; Demchuk et al., 2020; Nectow and Nestler, 2020; Wei et al., 2021; Kasatkina and Verkhusha, 2022; Liu et al., 2022; Robison and Nestler, 2022; Soutschek and Schratt, 2023). A sensory experience triggers a sensory-evoked activity, which drives a synaptic input onto the neurons and initiates membrane depolarization and calcium influx into the cytoplasm (Cohen and Greenberg, 2008). Different signaling pathways are involved in neural activity that triggers the expression of immediate early genes (IEGs), also called activity-dependent genes (Lee and Fields, 2021). For example, the Ca^2+^/CaMKII signaling pathway is triggered by calcium influx with the opening of voltage-sensing Ca^2+^ channels, NMDA receptors, and ryanodine receptors from the endoplasmic reticulum (extended data Figure 1). Following elevated intracellular calcium levels, calmodulin (CaM) binds to Ca^2+^, and CaMKII is activated to phosphorylate the cAMP response element-binding protein (CREB). Next, CREB binds to the cAMP response element and triggers the expression of IEGs (Deisseroth et al., 2003). Activity-dependent tools combine the proxies of neural activity, such as the increasing intracellular calcium levels, CREB phosphorylation, and expression of IEGs with different visualization and manipulation methods triggered by light or drug, including gene expression, uncaging light-sensing molecules, and photoswitching (Barykina et al., 2022). Most activity-dependent tools have a user-defined time window and can tag activated engram neurons via drugs or light; however, they cannot record the change in the neural activity longitudinally. A few researchers (Lin et al., 2023; Linghu et al., 2023) designed two new activity-dependent modular systems to report the occurrence of different cellular events at a certain time with different temporal resolutions, thereby adding a temporal dimension to studying complex physiological processes, such as memory formation and memory consolidation (Burgess, 2023; Kim et al., 2023; McNamara et al., 2023).

**Figure 1:**
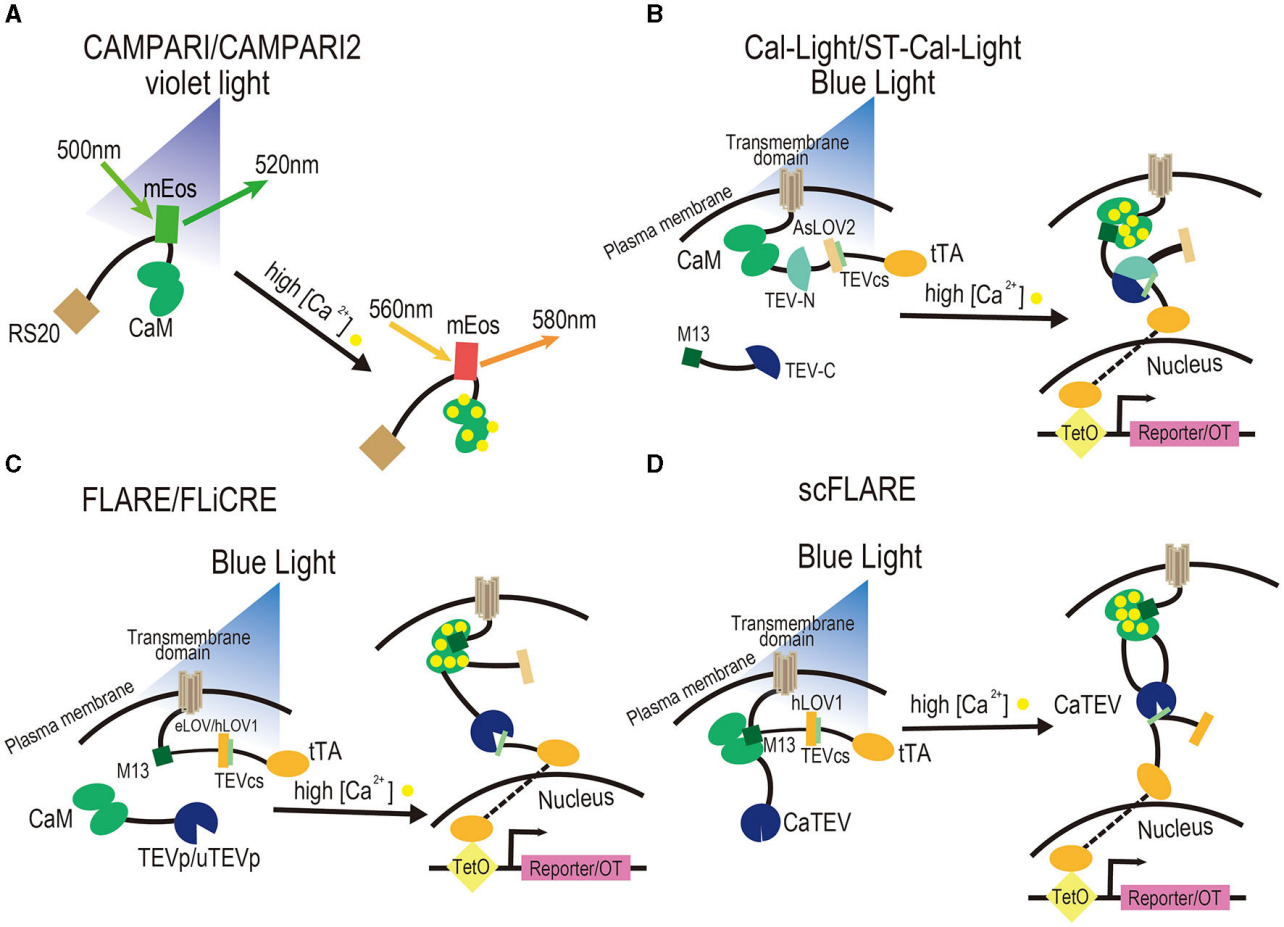
Design of activity-dependent tools based on Ca^2+^. **(A)** CaMPARI and CaMPARI2. They are photoconvertible fluorescent proteins that can be switched from green to red when illuminated with violet light and when intracellular calcium levels are elevated due to a stimulus. CaM and its binding peptide sense calcium levels through their interaction. **(B)** Cal-Light and ST-Cal-Light. They are tools that consist of two components and a reporter or optogenetics tool. The cytoplasmic component is M13 fused to the TEV-C, while the transmembrane component is a fusion of the transmembrane domain with CaM, the TEV-N, TEVcs caged within AsLOV2 domain, and tTA. To trigger the expression of a reporter or OT, it is necessary to elevate Ca^2+^ in the cytosol through a stimulus and illuminate blue light to cause a conformational change in the AsLOV2 to release TEVcs. Then, M13 and CaM bind to each other, TEV-C and TEV-N regain proteolytic functions to reconstitute TEVp, and the uncaged TEVcs are cleaved to drive the expression of tTA and further trigger the expression of a reporter or OT. **(C)** FLARE and FLiCRE. They contain two components and a reporter or OT. The cytoplasmic component is a fusion of CaM with TEVp, while the membrane component is a fusion of the transmembrane domain with the CaM-binding peptide M13, TEVcs caged within the eLOV domain, tTA, and a reporter/OT. To trigger the expression of a reporter or OT, it is necessary to elevate Ca^2+^ in the cytosol through a stimulus and illuminate blue light to cause a conformational change in the eLOV to release TEVcs. Then, M13 and CaM bind to each other, TEVp cleaves the uncaged TEVcs, and tTA is released to drive the expression of a reporter or OT. FLiCRE is an optimized version of FLARE, which contains TEVp with a faster turnover rate and a version of LOV domain with tighter caged TEVcs named hLOV1. **(D)** scFLARE. It consists of a membrane component and a reporter. The membrane component consists of a transmembrane domain, engineered CaTEV, TEVcs caged within the engineered hLOV1, and tTA. To trigger the expression of a reporter or OT, it is necessary to elevate Ca^2+^ in the cytosol through a stimulus to activate CaTEV and illuminate blue light to cause a conformational change in the hLOV1 to release TEVcs. Then, CaTEV cleaves the TEVcs, releases tTA, and drives the expression of a reporter or OT. All FLARE-derived systems contain soma-targeting signals in their membrane components.

In this review, we mainly describe the background, principle, and selected examples of applications of the existing activity-dependent tools to label and manipulate engram neurons, including calcium-based activity-dependent tools, CREB regulating systems, and IEG-based activity-dependent tools (Table 1). In addition, we have reviewed activity-dependent tools for longitudinal records of cellular events. At last, we propose certain limitations encountered while using these tools. We look forward to the future development and potential of activity-dependent tools.

**Table 1:** Summary of the tools mentioned in the review.

**Tools**		**Key elements**	**Applicability**	**Compatibility (opto- or chemo-genetic tools)**
Calcium-based activity-dependent tools	CaMPARI	mEos and CaM	Mice, zebrafish, and cultured neurons	Incompatibility
	CaMPARI2	mEos and CaM	Mice, zebrafish, and cultured neurons	Incompatibility
	rsCaMPARI	mEos and CaM	Zebrafish	Incompatibility
	Cal-light	Inducible Tango system and CaM	Mice and cultured neurons	High compatibility
	ST-Cal-light	Inducible Tango system and CaM	Mice and cultured neurons	High compatibility
	FLARE	Inducible Tango system and CaM	Mice and cultured neurons	High compatibility
	FLiCRE	Inducible Tango system and CaM	Mice and cultured neurons	High compatibility
	scFLARE	Inducible Tango system	cultured neurons	High compatibility
CREB regulating system		CREB	Mice and cultured neurons	Only the Opto-DN-CREB system has high compatibility with optogenetic tools now.
Chemical-based activity-dependent tools based on immediate early genes	TetTag	Tet-Off system and doxycycline	Mice	High compatibility
	TRAP	Cre^ERT^ and tamoxifen	Mice	High compatibility
	LacZ-Daun02	lacZ and Daun02	Rats	Incompatibility
	CANE	DsTVA and EnvA	Mice	High compatibility
	vGATE	Tet-On system and doxycycline	Mice	High compatibility
	TRACE	AAV2-retro and Cre^ERT^	Mice	High compatibility
	E-SARE	Arc promoter	Mice and cultured neurons	High compatibility
	RAM	Fos promoter	Mice	High compatibility
Activity-dependent tools for longitudinal records of cellular events	iPAK4 strategy	iPAK4, baseline timestamps, and reporter gene	Cultured neurons	Incompatibility
	XRI strategy	1POK (E239Y), maltose-binding and protein epitope tag	Mice and cultured neurons	Incompatibility

## 2 Calcium-based activity-dependent tools

### 2.1 Background

Single-component fluorescent calcium integrators have emerged as a powerful tool to map activated neural circuits due to their faster response to neural activity compared to the IEG-based systems that are limited by drug delivery and metabolic speed in the brain (Greenwald et al., 2018; Losi et al., 2018; Luo et al., 2018; Mena et al., 2018; Robinson and Gradinaru, 2018; Wang et al., 2018; Lin et al., 2019; Abreu and Levitz, 2020; Chung and Lin, 2020; Labouesse et al., 2020; Covey and Yocky, 2021; Pearce and Tucker, 2021; Gao et al., 2022; Manhas et al., 2022; Shen et al., 2022; Wu et al., 2022). These integrators comprise photoconvertible or photoswitchable proteins, also called genetically encoded calcium indicators (GECIs; Mank and Griesbeck, 2008; Grienberger and Konnerth, 2012; Looger and Griesbeck, 2012; Kaestner et al., 2014; Gibhardt et al., 2016; Lin and Schnitzer, 2016; Suzuki et al., 2016; Zhong and Schleifenbaum, 2019; Broussard and Petreanu, 2021; Inoue, 2021), which bind to calcium and undergo a rapid switch following exposure to light.

GECIs lack the manipulating function, a limitation that was overcome by the development of Cal-Light by Lee et al. (2017) and FLARE (fast light- and activity-regulated expression) by Wang et al. (2017). Cal-Light and FLARE provided new tools to conduct more detailed studies on neurons. These tools are based on the modular gene expression structure of the protein interaction system, Tango (Barnea et al., 2008; Kroeze et al., 2015), which monitors the activity of G protein-coupled receptors (GPCRs), receptor tyrosine kinases, steroid hormone receptors, and neuromodulator receptors. Moreover, the calcium-based detection of GECIs and photoreactive properties of the light-oxygen-voltage (LOV; Harper et al., 2003) domain are used to achieve photo-controlled calcium ion-dependent active neural ensemble markers and target gene expression with a high temporal and spatial resolution.

### 2.2 CaMPARI/CaMPARI2/rsCaMPARI

#### 2.2.1 Principle

Calcium-modulated photoactivatable ratiometric integrator (CaMPARI) is a calcium-modulated photoactivatable ratiometric integrator that utilizes GECIs. Its functioning was first demonstrated by Fosque et al. (2015). CaMPARI is derived from the circularly permuted photoconvertible fluorescent protein mEos (Matos et al., 2019). Following brief UV irradiation, mEos, which is allosterically regulated and fluoresces green upon calcium binding, is converted to a red fluorescent product, providing high temporal and spatial resolution labeling of active neurons. CaM and CaM-binding RS20 peptide (Mirzoeva et al., 1999) are attached to the N- and C-termini of mEos, respectively. The measurement of red fluorescence correlates with neuronal activity (Figure 1A). The time window of CaMPARI is shorter than that of IEG-based activity-dependent tools based on its photoconversion rate.

CaMPARI2 is an upgraded version of CaMPARI with a slower photoconversion rate in the calcium-free state. Thus, it offers a higher contrast in green-to-red photoswitching between calcium-bound and calcium-free states. This is achieved through saturated mutagenesis of amino acid positions surrounding the chromophore and in the interface between the fluorescent and calcium-binding domains of CaMPARI (Moeyaert et al., 2018). CaMPARI2 exhibits rapid calcium unbinding kinetics in cultured neurons, providing better temporal resolution and decreasing noise in low calcium conditions.

Reversibly switchable CaMPARI (rsCaMPARI) is a calcium integrator that can be reversibly switched from a bright to a dark state using a combination of blue light and elevated calcium levels. Thus, it can be switched from a dark to a bright state by violet light illumination (Sha et al., 2020). The rate of switching off is significantly faster in the calcium-bound state than in the calcium-free state, making rsCaMPARI a negative response indicator.

As a reversibly photoswitchable fluorescent protein, the functioning of rsCaMPARI is limited by photofatigue, resulting in a loss of brightness during multiple cycles of photoswitching. The rsCaMPARI can be subjected to 10 cycles of switching in the neurons, following which the contrast between the calcium-free and calcium-bound states decreases to less than a 2-fold difference (Lee et al., 2017; Sha et al., 2020).

#### 2.2.2 Selected examples of applications

CaMPARI has been reported to be stably expressed in mammalian brains (Ebner et al., 2019) and transgenic zebrafish larvae (Fosque et al., 2015). However, CaMPARI is most effective in organisms penetrable by light. A study reported that CaMPARI enabled two-photon calcium imaging of active neurons in layer 2/3 of the mouse visual cortex in response to moving gratings with the *post hoc* staining of visual cortex orientation maps over a large brain volume (Fosque et al., 2015). Another study demonstrated that the CaMPARI permanent markers could measure signals from large areas of tissue and can easily correlate the activity with other structural or functional tags. CaMPARI can label neurons as specific groups of postsynapses for photogenetic stimulation, resulting in all-optical functional connectivity mapping (Zolnik et al., 2017). Nevertheless, CaMPARI has certain limitations. UV irradiation can exert a harmful effect, and it is not possible to introduce manipulative elements into the activated neurons for functional analysis.

Compared to its original version, CaMPARI2 displays higher molecular brightness in its red form, as demonstrated by *in vitro* screening assay and zebrafish experiments (Moeyaert et al., 2018). This tool has been validated for labeling neural circuits in response to visual stimuli in the mouse visual cortex and in the whole brain of transgenic larval freely swimming zebrafish (Moeyaert et al., 2018). Interestingly, other studies targeting CaMPARI and CaMPARI2 indicated that the red–green ratio of CaMPARI in the active neurons was significantly higher than that of CaMPARI2. In addition, CaMPARI functions as a more sensitive conventional Ca^2+^ sensor than CaMPARI2, producing more changes in active-driven dynamic fluorescence in the *in vivo* experiments (Das et al., 2022).

rsCAMPARI is primarily used for studying neural circuits in zebrafish. It has been used to label active neural circuits in freely swimming transgenic zebrafish (Fosque et al., 2015; Sha et al., 2020). No study on its use in understanding engram cells has been published.

### 2.3 Cal-Light/ST-Cal-Light

#### 2.3.1 Principle

Cal-Light (Lee et al., 2017), a two-component system, replaces GPCR and β-arrestin in the inducible Tango system (Lee et al., 2017) with CaM and M13/M2 (a CaM-binding peptide). The components of Cal-Light include a calmodulin-binding peptide (M13) fused to the C-terminus of split-tobacco etch virus protease (TEVp; TEV-C), a fusion of the transmembrane domain with CaM, the N-terminus of split TEVp (TEV-N), a caged TEV cleavage site (TEVcs) within the Jα helix of the AsLOV2 domain, and tetracycline-controlled transactivator (tTA). In the dark, the TEVcs located at the C-terminus of the Jα helix of AsLOV2 are inaccessible due to steric hindrance (Figure 1B). Following blue light illumination, the Jα helix with TEVcs is released from the AsLOV2 domain, and TEVcs are cleaved by TEVp. When intracellular calcium ion levels increase, CaM binds to it and initiates conformational changes to attract M13/M2, bringing the two separated structural modules closer. Blue light irradiation induces conformational changes in LOVs and exposes the TEVp restriction site. Next, the TEVp restriction reaction releases tTA from the membrane, which enters the nucleus to initiate the expression of the target component and achieve photo-controlled Ca^2+^-dependent active labeling or manipulation. To minimize the background, a C-terminally truncated version of TEVp with reduced affinity to TEVcs was used in Cal-Light. However, Cal-Light demonstrated a certain background in light/no activity conditions, which could be attributed to the self-reconstitution of split TEVp fragments (Kim et al., 2017). The reversibility of the split TEVp was slow (20–60 min; Kim et al., 2017), which limited the temporal resolution of the system.

ST-Cal-Light (soma-targeted version of Cal-Light) is an improved version of Cal-Light. To increase the signal-to-noise ratio, researchers inserted a kainate receptor subunit 2 soma-targeting peptide between the cytosolic side of the transmembrane domain and CaM, restricting the system to the cell body (Hyun et al., 2022; Figure 1B). ST-Cal-Light was checked in neuronal cultures and displayed a 1.8 to 2-fold higher signal-to-noise ratio than the Cal-Light system *in vitro* (Hyun et al., 2022).

#### 2.3.2 Selected examples of applications

In a study involving lever-pressing training in mice, researchers used Cal-Light to label the activated neural ensembles in the primary motor cortex (Kim et al., 2017). Mice were exposed to blue light to drive the expression of the inhibitory opsin eNpHR in the learning-related neural ensembles *in vivo*. Specifically, blue light was delivered for 5 s during each of the 11 lever presses in 45-min sessions when water-restricted mice learned to press the lever to obtain a water reward. Afterward, yellow light was used to successfully inhibit the neuronal activity of the labeled neurons, as verified by electrophysiology. This intervention suppressed the lever-pressing behavior, whereas locomotivity in freely behaving mice remained unaffected. Another study used ST-Cal-Light to label and suppress the activity of neural ensembles in the medial prefrontal cortex that was involved in social behavior by light-gating the inhibitory opsin eNpHR (Roy et al., 2016). Furthermore, knock-in ST-Cal-Light mice were created to reduce the impact of infectious efficiency of related viruses. These findings suggest that Cal-Light or ST-Cal-Light has great potential in applying to the field of neurobiology.

### 2.4 FLARE, FLiCRE, and scFLARE

#### 2.4.1 Principle

FLARE is a tool that combines calcium and light sensing to identify neural ensembles active during a specific time window. Similar to Cal-light, it contains a fusion of CaM with TEVp and a transmembrane domain with a soma localization sequence, CaM-binding peptide, TEVcs caged within the Jα helix of the evolved LOV (eLOV) domain, and tTA (Wang et al., 2017; Figure 1C). However, compared to similar systems, the caging of TEVcs in FLARE is more efficient due to the direct molecular evolution in yeast (Sanchez and Ting, 2020). Calcium sensitivity is achieved through interaction between CaM and a CaM-binding peptide that brings TEVp close to TEVcs. A C-terminally truncated version of TEVp is used to minimize TEVc cleavage by cytoplasmic TEVp without calcium elevation. This version of TEVp has low substrate affinity, favoring proximity-dependent cleavage. Following neuronal activity and blue light stimulation, tTA is released and initiates the expression of reporter or effector genes (Wang et al., 2017). Thus, when FLARE detects an increase in calcium ions and a blue light signal, it drives tTA into the nucleus, initiating the expression of a fluorescent reporter protein or effector gene, allowing researchers to identify the activated neurons for manipulation.

Compared to FLARE, FLiCRE (fast light and calcium-regulated expression) uses an ultra-fast TEVp variant with a higher turnover rate than the original TEVp, along with an improved version of a LOV domain featuring a tighter caged TEV cleavage site (Kim et al., 2020). This improved LOV domain is called the hybrid LOV domain (hLOV1; Kim et al., 2020; Figure 1C). A similar system, FLARE2, was also developed, which enhances the kinetics of TEVp cleavage, resulting in a shorter time window compared to FLARE (Sanchez and Ting, 2020).

To simplify the two-component light-gated systems and reduce their performance dependence on the interaction between them, scFLARE (single-chain FLARE) was designed (Sanchez et al., 2020). scFLARE combines modules responsible for calcium-sensing, light-sensing, peptide cleavage, and transcription activation in a single polypeptide chain. The system's key component is a calcium-dependent TEV protease (CaTEV), consisting of a calcium-sensing module comprising CaM and a CaM-binding peptide (M13) combined with one of the exposed loops of TEVp. This configuration allows regulation by calcium (Sanchez et al., 2020), as illustrated in Figure 1D. Similar to FLARE and FLiCRE, with the coincidence of blue light and an increase in calcium levels, CaTEV is activated, cleaving the uncaged TEVcs and releasing tTA to promote the expression of target genes.

#### 2.4.2 Selected examples of applications

FLARE was first validated by Wang et al. (2017) as a combination with optogenetics. Scientists injected the FLARE into the motor cortex of adult mice via virus and stimulated the mice by wheel running. Significantly elevated expression of reporter genes was noted in the motor cortex of mice running during the blue light period than in those inactive and in the absence of blue light.

Scientists used the FLiCRE system to identify a specific neuronal subtype in the NAc that was activated by the local stimulation of upstream ChR2-expressing axons from excitatory glutamatergic neurons (Kim et al., 2020). In this study, neural ensembles in the VTA regions were activated by acute nicotine administration to increase intracellular Ca^2+^ levels. Next, blue light was delivered to trigger the expression of mCherry genes in FLiCRE. This approach expanded the application of FLiCRE in biological studies.

Although scFLARE has only been validated in *in vitro* experiments (Sanchez and Ting, 2020), it harbors great potential for biological research because of its more robust functions than FLARE.

### 2.5 Summary of calcium-based activity-dependent tools

Based on the change in calcium levels and light gating, the primary advantage of these tools is their very short time window and rapid response to neural activity (Barykina et al., 2022). Without inducing the expression of reporter genes, CaMPARI, and its improved versions have faster responses to the change in calcium concentrations and indicate neural activity compared to other tools mentioned in this chapter. However, the use of ultraviolet light limits its application due to its poor safety (Begovic et al., 1991; Shah et al., 2005; Maverakis et al., 2010; Craig et al., 2018; Neubert et al., 2019; Liu C. et al., 2020). The use of different wavelength light is vital as it determines the safety of experimental subjects and researchers as well as the penetrability of the tissue (Sutton, 1993; Ai et al., 2016; Yang et al., 2017; Dash et al., 2021; Huang, 2022). Cal-Light and FLARE, developed according to the inducible Tango system (Lee et al., 2017), have enough components with genetic strategies to tag and manipulate engram cells in a short user-defined time window. However, their high sensitivity to calcium demands complete controlled trials to study the artifact caused by the background. Moreover, using these tools only via virus could result in variable expression of constructs and systems (Barykina et al., 2022). Therefore, constructing transgenic mice or rats and decreasing the components, such as the scFLARE system, could be one of the future directions. A new tool using a calcium-dependent luciferase to convert neural activity into the activation of light-sensing domains within the same cell can tag and manipulate active neurons using genetic strategies. The new tool has been validated in individuals and populations both *in vivo* and *in vitro*, thus providing novel ideas to develop activity-dependent tools combined with enzyme induction and light gating (Crespo et al., 2023).

## 3 CREB regulating system

### 3.1 Background

CREB regulates the gene expression of several biological processes (Sheng et al., 1991; Silva et al., 1998; Tanis et al., 2008; Yamaguchi and Hearing, 2009; Raefsky and Mattson, 2017; Saura and Cardinaux, 2017; Awasthi et al., 2021; Hua et al., 2021; Lin et al., 2021; Albarnaz et al., 2022; Mi et al., 2022). For example, CREB governs the expression of IEGs and is substantially involved in the memory allocation process and selective recruitment of its coding. Researchers have developed multiple CREB regulating systems to study underlying mechanisms and biological functions (Silva et al., 1998; de Armentia et al., 2007; Hwang et al., 2011; Ha et al., 2014; Karelina et al., 2015; Zhang et al., 2016; Caracciolo et al., 2018; Ahmed et al., 2022; Mi et al., 2022; Chuan et al., 2023).

### 3.2 Principle

CREB is present in almost all promoter regions of IEGs and is considered a core factor regulating the expression of IEGs. For example, when neurons are activated by experience, the phosphorylated form of CREB binds to the 5′ non-coding regulatory region of the *c-Fos* gene, upregulating the expression of c-Fos (Scott et al., 2002). Thus, CREB couples experience-dependent neuronal activation with gene transcription and long-term cellular and molecular changes associated with plasticity, learning, and memory (Barth et al., 2000). Han et al. (2007) combined the CREB gene sequence and Cre-loxP system carried by a virus to label and manipulate engram neurons overexpressing CREB. To endogenously mediate the CREB's activity *in vivo* (Zhu et al., 2004), two dominant-negative mutants of CREB, termed CREB-S133A and CREB-R287L, and a constitutively active CREB called VP16-CREB, were introduced. They were constructed by changing a specific site necessary for endogenous CREB binding to targeted genes. Similarly, Ali et al. (2015) developed a new CREB regulating system using a combination of CREB and optogenetics called Opto-DN-CREB. It is a blue light-controlled inhibitor of this component, which fuses the dominant negative inhibitor A-CREB to photoactive yellow protein and controls the expression of CREB in the neurons, both spatially and temporally.

### 3.3 Selected examples of applications

Hsiang et al. (2014) used ablation or silencing techniques on neurons overexpressing CREB to identify the specific neural circuits associated with cocaine-induced engrams that support associative memory for cocaine cues. Their results demonstrated that neurons with elevated levels of CREB were preferentially recruited or assigned to cocaine-associated cues, suggesting their critical role in creating a larger cocaine memory engram. Similarly, Rao-Ruiz et al. (2019) performed unbiased RNA sequencing on DG engram neurons 24 h after contextual fear conditioning to identify memory consolidation-specific transcriptional changes. They identified highly distinct gene expression patterns in DG engram neurons, with prominent CREB and Kcnq3-dependent transcriptional features. They used the mCREB component to inhibit the endogenous expression of CREB, also called CREB-S133A, and validated the functional relevance of RNAseq findings by establishing a causal relationship between intact CREB function in DG imprints during memory consolidation. Furthermore, researchers have used the Opto-DN-CREB system to randomly select a small population of LA neurons to investigate the impact of rapidly increased CREB levels during the 1st min before and after fear conditioning on supporting engram neurons, memory consolidation, and retrieval for auditory fear conditioning. They demonstrated that the timing of CREB activation is critical for memory expression in relation to the training afterward (Park et al., 2020).

### 3.4 Summary of CREB regulating system

The CREB regulating system was designed differently to regulate the activity of CREB. Initially, a plasmid was used as an ideal vector to carry the CREB overexpression system and was widely used in cultured cells (Chen et al., 2014). For *in vivo* experiments, viruses have been used to carry the genetic elements related to CREB to replace plasmids as a carrier. In addition, exogenous expression of CREB has been used to mediate neuronal allocation (Han et al., 2007). Because CREB is involved in several biological processes, exogenously augmenting its level could affect the physiological stages of experimental animals (Newton and Dixit, 2012; Elton et al., 2013; Steven and Seliger, 2016; Belgacem and Borodinsky, 2017; Fidaleo et al., 2017; Chen et al., 2019; Sen and Stress, 2019; Nouri et al., 2020; Tang et al., 2020; Benchoula et al., 2021; Tropea et al., 2022). To solve this problem, endogenous genetic components, such as CREB-S133A, CREB-R287L, and VP16-CREB, have been constructed to induce the expression of CREB (Zhu et al., 2004). To improve the temporal and spatial resolutions of the CREB regulating system, novel genetic strategies, such as optogenetics and tamoxifen-induced gene expression system (Kida et al., 2002), have been combined with CREB-mediated components to more accurately induce the expression of CREB. The development of molecular biological technology and other convenient methods, for example, small interfering RNAs (Manoharan, 2004; Kanasty et al., 2013; Nambudiri and Widlund, 2013; Hong and Nam, 2014; Alshaer et al., 2021) and CRISPR/Cas9 (Ma et al., 2014; Hryhorowicz et al., 2017; Gupta et al., 2019; Banan, 2020; Horodecka and Duchler, 2021) technologies, have ushered in a new potential for the regulation of CREB's activity and have provided scientists with more options to study its mechanisms in biological processes and diseases (Saura and Valero, 2011; Li et al., 2018; Won et al., 2019; Sharma and Singh, 2020; Wang et al., 2021; Cui et al., 2022; Tropea et al., 2022; Yao et al., 2022; Zhang et al., 2023).

## 4 Chemical-based activity-dependent tools based on immediate early genes

### 4.1 Background

Identifying activated neurons constituting functional circuits on a large spatial scale within a limited time frame necessitates accurately correlating the circuit dynamics with perceptual, cognitive, affective, and motor functions in behavior (Selverston, 1992; Wilson and McNaughton, 1994; Lewis and Eisen, 2003; Boyce and Mendell, 2014; Gibson et al., 2014; DeNardo and Luo, 2017; Marachlian et al., 2018; Sheng et al., 2018; Liu D. et al., 2020; Maluck et al., 2020). In this regard, IEG expression is used as a proxy for neural activity.

To integrate chemicals and IEG expression resulting from neuronal activity, components of drug-dependent gene expression systems are combined with IEG promoters. The readout of these systems involves the expression of reporter genes or effector genes, which can label or manipulate previously activated neurons.

### 4.2 TetTag

#### 4.2.1 Principle

Reijmers et al. (2007) developed the tetracycline tag system (TetTag) using the Tet-Off conditional gene expression system (Lewandoski, 2001). This innovation enables the first long-term labeling of active neural ensembles within a specific time interval.

**Figure 2:**
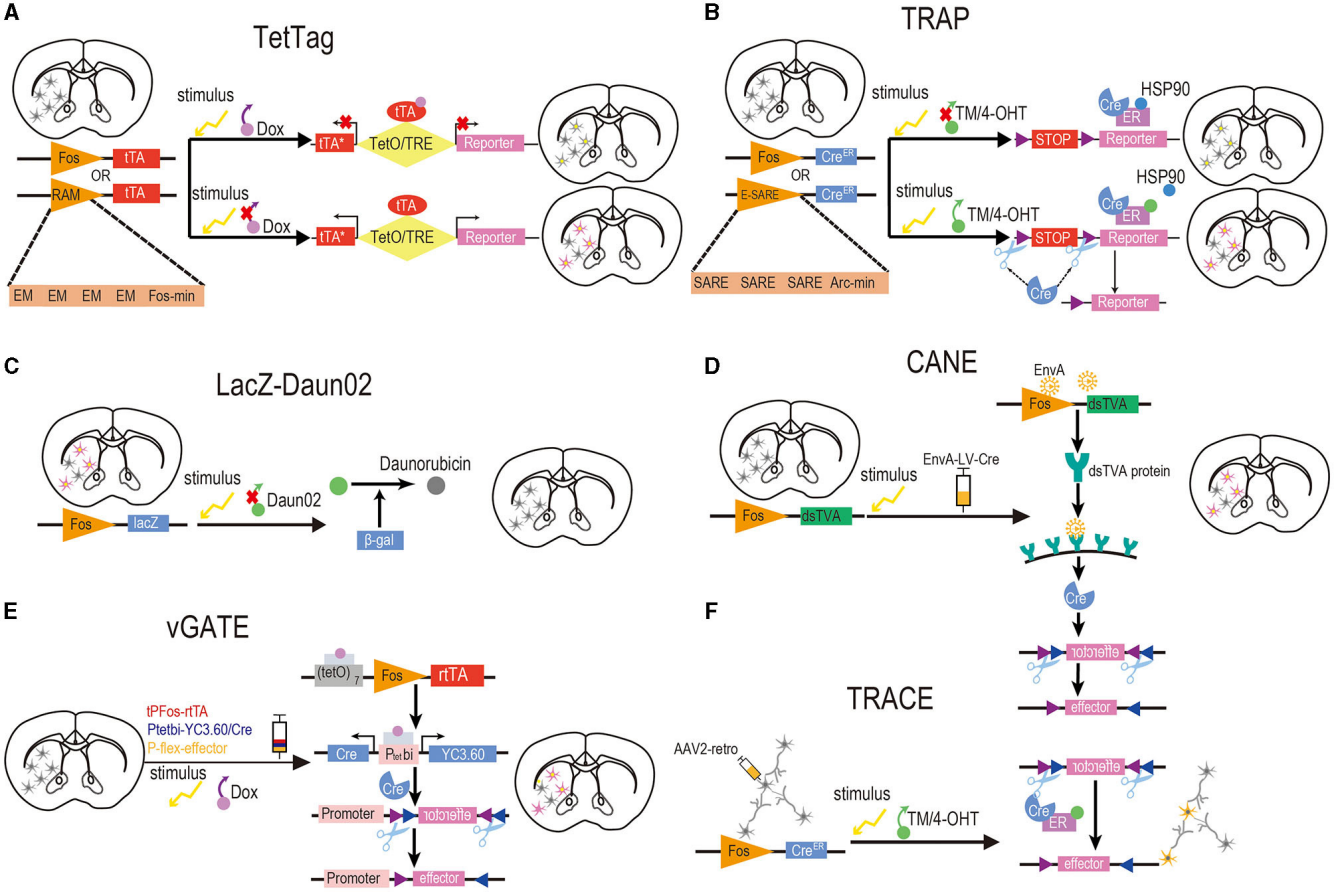
Design of activity-dependent tools based on chemicals and IEGs. **(A)** TetTag. To induce reporter gene expression in target neurons, doxycycline must be removed from the food of Fos-tTA mice, and a specific stimulus should be provided concurrently to initiate Fos expression, resulting in tTA expression. Controlled by tetracycline-responsive elements under the regulation of the TetO, tTA drives the expression of the reporter genes. RAM. The RAM synthetic promoter comprises four tandem repeats of synthetic EM located upstream of the minimal Fos promoter. Commonly employed in conjunction with TegTag, the RAM promoter effectively drives the expression of destabilized tTA to initiate the production of a reporter. **(B)** TRAP and TRAP2. For the expression of reporter genes in activated neurons, it is essential to administer TM or 4-OHT to Fos-CreERT2 mice, while concurrently providing a specific stimulus to initiate Fos expression. The expression of CreERT2, in turn, drives the expression of Cre-dependent reporter genes. E-SARE. In this synthetic promoter, the SARE enhancer is fused to the minimal Arc promoter in five tandem repeats. It is usually used with the TRAP tools and drives the Cre recombinase effectively to trigger the expression of reporter genes. **(C)** LacZ-Daun02. To selectively inactivate neurons activated by stimulus, c-fos-lacZ rats were designed to label activated neurons expressing both c-Fos and the β-gal encoded by the lacZ gene. After providing a specific stimulus, precursor drug Daun02 is injected into specific brain regions where β-gal catalyzes the conversion of Daun02 to daunorubicin. This drug could inactivate neurons activity. So, neurons expressing β-gal and c-Fos can be selectively inactivated in this way. **(D)** CANE. To induce reporter gene expression in activated neurons, it is essential to provide a specific stimulus to initiate Fos expression and inject lentiviruses coated with an EnvA into FosTVA mice. Subsequently, the Fos promoter will stimulate the expression of dsTVA, which can bind a coat protein of EnvA. The EnvA-RV-infected neurons will then deliver the Cre recombinase that drives the expression of reporter genes. **(E)** vGATE. Prior to initiating the experiment, it is required to inject three components (tPFos-rtTA + Ptetpi-YC3.60/Cre + POT-flex-effector) packaged by AAV into WT mice. To induce reporter gene expression in activated neurons, a specific stimulus must be provided to initiate Fos expression, and Dox should be added to the food of WT mice to activate rtTA. The Fos promoter drives the expression of Dox-activated rtTA, which is further autoregulated in a loop manner. Both reporter genes and Cre recombinase are expressed by rtTA, which subsequently induces the expression of the effector. **(F)** TRACE. The TRAP method is combined with the expression of a reporter gene from the AAV2 retrograde virus. This approach labels activated neurons in the target region and afferent neurons from other regions with the reporter gene. Yellow neurons in **(F)** indicate successful activation and labeling, while gray neurons in **(F)** denote inactivation. In brain slices, neurons with yellow nuclei and purple cytoplasm signify successful activation and labeling, whereas neurons with yellow nuclei and gray cytoplasm represent activation without successful labeling. Neurons in brain slices that are gray indicate inactivation.

The TetTag system combines the expression of tetracycline- (or its analog doxycycline) dependent tTA protein with the activation of the Fos promoter (Reijmers et al., 2007). Following stimulation, activated neurons initially drive tTA expression via the c-Fos promoter, allowing it to bind to the tetracycline response element and regulate target gene expression. In the presence of Dox, the expression of tTA from the Fos promoter during neuronal activity is inhibited, preventing the activation of the reporter gene (Figure 2A).

#### 4.2.2 Selected examples of applications

Initially, TetTag mice were used to tag a neural ensemble in the basolateral amygdala activated during learning and subsequently reactivated during the retrieval of fear memory (Reijmers et al., 2007). In addition, scientists employed this system to label and manipulate the activated engram cells in the core of the nucleus accumbens and vCA1 regions during re-exposure to the conditional-placed-preference apparatus to assess whether the retrieval of cocaine-induced CPP memory occurred through the activation of engram cells in these two regions using chemogenetic tools (Zhou et al., 2019). Recently, Kuner et al. identified a small group of neurons in the prefrontal cortex (PL) of the brain that modulates the interaction between long-term fear memory and pain (Stegemann et al., 2023). The researchers have used the Tet-Off system to specifically label prefrontal engrams in long-term fear and pain cells active in limited time windows and further identified that a small group of neurons in the PL overlap between fear memory and pain.

### 4.3 TRAP

#### 4.3.1 Principle

Targeted recombination in active populations (TRAP) represents the first permanent labeling of activated neural ensembles. Researchers generated FosTRAP or ArcTRAP mice by inserting CreERT2 into the c-Fos and Arc promoters, respectively, and tagged active neural ensembles at specific time intervals by combining Cre-dependent reporter genes or effector genes (Guenthner et al., 2013; Figure 2B). CreERT2 is driven by c-Fos promoters and sequestered in the cytoplasm. CreERT2 is released following the administration of tamoxifen (TM) or its metabolite 4-hydroxytamoxifen (4-OHT), allowing Cre recombinase to translocate into the nucleus and mediate recombination. This activates the expression of target genes in activated neural ensembles within a defined time window (Feil et al., 1997).

#### 4.3.2 Selected examples of applications

TRAP has been used to demonstrate the role of the hippocampus engram in long-term memory. Denny et al. designed the ArcCreERT2 bacterial artificial chromosome (BAC) transgenic mice to understand how a memory trace is generated and retrieved in the hippocampus under different conditions (Denny et al., 2014). Clawson et al. used TRAP to genetically label or optogenetically manipulate the primary visual cortex engram neurons responsive to the visual cue and found that the neurons are selectively reactivated during post-conditioning sleep (Clawson et al., 2021).

### 4.4 LacZ-Daun02

#### 4.4.1 Principle

Until 2009, no methods were available to manipulate the activated neurons and establish their function in behavioral effects and cues. To address this issue, researchers introduced the Daun02 inactivation method, which selectively inactivates previously activated neural ensembles (Koya et al., 2009). The system comprises two components (Figure 2C). The first component is c-fos-lacZ transgenic rats carrying a transgene consisting of a c-fos promoter containing the bacterial lacZ gene encoding β-galactosidase protein. Stimulation of the c-fos promoter induces the expression of β-galactosidase in the activated neurons, which can be detected using X-gal staining (Kwon and Houpt, 2010). The second component is Daun02, a prodrug that can be converted to daunorubicin by β-galactosidase, reducing calcium ion-dependent action potentials in neuroblastoma cells (Andrés et al., 1996). Consequently, Daun02 can selectively inactivate neural ensembles expressing β-gal following its injection into target regions. Moreover, the extent of inactivation can be assessed through X-gal staining of β-gal expression.

#### 4.4.2 Selected examples of applications

Koya et al. (2009) selectively inactivated cocaine-activated neurons in a setting paired with repeated drug injections using the LacZ-Daun02 inactivation system. They identified small subpopulations of neurons in the nucleus accumbens selectively activated by cocaine in specific contexts while mediating context-specific psychomotor sensitization (Koya et al., 2009). Similarly, Kane et al. (2021) induced Fos and β-gal expression in rats exposed to food- and cocaine-seeking conditions, respectively, and subsequently inactivated the β-gal expressing neural ensembles by injecting Daun02 into the ventral medial prefrontal cortex. The neural ensembles activated by cocaine seeking in the ventral medial prefrontal cortex were functionally distinct from those activated by food seeking. Furthermore, the lacZ-Daun02 inactivation system has been used to investigate the mechanisms underlying behavioral sensitization (Koya et al., 2009), craving (Fanous et al., 2012; Pfarr et al., 2015; Funk et al., 2016; Caprioli et al., 2017), and relapse induced by addictive drugs.

Lay et al. (2023) microinjected Daun02 in Fos-lacZ transgenic rats following a single extinction training episode. They reported that the initial extinction-recruited central nucleus of the amygdala (CN) ensemble was critical to the acquisition-extinction balance and that greater behavioral restoration did not imply weaker extinction contribution. The researchers used this technique to delete the extinction-recruited neuronal ensembles in the basolateral amygdala and CN and examined their contribution to behavior in an appetitive Pavlovian task. Subsequently, they demonstrated that the deletion of these extinction-activated ensembles in the CN impaired the retrieval of extinction.

### 4.5 CANE

#### 4.5.1 Principle

The capturing activated neural ensembles (CANE) system is based on the destabilized TVA (dsTVA, analytically targeted avian leukemia) receptor and leukosis virus (EnvA, a bird-specific virus), which was developed by Sakurai (Sakurai et al., 2016; Figure 2D). To avoid receptor accumulation resulting from the previous neuronal activity, dsTVA is fused to the PEST degradation sequence. FosTVA mice are generated by inserting the 2A-dsTVA construct into the endogenous Fos locus. EnvA-coated rabies virus (EnvA-RV) or lentivirus (EnvA-LV) is stereotaxically injected into the target brain regions of FosTVA mice to infect the TVA-expressing neurons and deliver Cre effectors, which mediate the expression of reporter genes for permanent neuronal labeling.

#### 4.5.2 Selected examples of applications

CANE has been used to demonstrate that social fear and aggression activate distinct, mostly non-overlapping neural ensembles in the hypothalamus of mice (Sakurai et al., 2016). Neural circuits activated during the studied behavior were labeled. A combination of CANE and optogenetic tools was used to manipulate the social fear neurons in the hypothalamus. Optogenetic activation of social fear neural circuits was sufficient to elicit fear-like behavior in the absence of fear. In addition, CANE can be used to identify previously unidentified monosynaptic connections between cranial sensory neurons and nociceptive neurons of the parabrachial nucleus (Kane et al., 2021).

### 4.6 vGATE

#### 4.6.1 Principle

Scientists have developed virus-delivered genetic activity-induced tagging of cell ensembles (vGATE) based on the Tet-On system. It consists of three AAV components (Baron and Bujard, 2000; Figure 2E). In the first component, neural activity drives rtTA expression through the Fos promoter within an autoregulatory expression loop, causing its sustained expression in a Dox-dependent manner. This loop is designed by incorporating the rtTA-binding tetracycline operator (TetO) sequences [(TetO)7] upstream of the Fos promoter. In the second component, the Dox-dependent bidirectional tet promoter controls the expression of a reporter gene and Cre recombinase. In the third component, cell-specific promoters control the expression of effectors of interest in a Cre-dependent manner (Hasan et al., 2019).

#### 4.6.2 Selected examples of applications

Hasan et al. (2019) developed vGATE to tag a context-specific fear memory engram in the hypothalamic oxytocin system that participated in rapid unfreezing, extinction, and enhanced glutamatergic transmission. The time window of vGATE is determined by Dox injection and metabolism in the brain, and intraperitoneal injection of Dox allows precise temporal control and avoids dependency on animal diet.

### 4.7 TRACE

#### 4.7.1 Principle

TRACE is an unbiased approach used to label afferent inputs specifically activated by a defined stimulus in an activity-dependent manner (Krauth et al., 2020). This method is based on two components: the TRAP system, which was mentioned previously, and a retrograde virus (AAV2-retro) carrying Cre-dependent reporter genes to label the activated neurons in specific neural circuits. The Arc-CreERT2 and Fos-CreERT2 transgenic mice, also used in the TRAP system, are used to explore neural circuits with the TRACE method. To use TRACE, AAV2-retro is injected into the brain of transgenic mice and allowed to infect the target regions and axons of the projecting neurons. Once the animals are exposed to a specific behavioral experience, 4-OHT or TM is injected to induce the translocation of the CreERT2 recombinase to the nucleus in the activated neurons that experienced the behavior. These neural ensembles can be permanently labeled by reporter genes carried by AAV2-retro in a Cre-dependent manner (Figure 2F).

#### 4.7.2 Selected examples of applications

Researchers have used The TRACE system to specifically label and investigate different behaviorally relevant neural circuits (Nabavi et al., 2014). For instance, scientists used TRACE to label neurons mediating high-frequency stimulation from the temporal association area and ectorhinal cortex to the lateral amygdala, a well-established pathway with behavioral significance (Kawashima et al., 2013). This research has paved the way to further understand the engram across memory stages and reveal the logic of memory in the brain.

### 4.8 E-SARE and RAM synthetic promoters

Compared to the natural IEG promoters, synthetic active promoters E-SARE and RAM have been successfully developed to enhance the effectiveness and performance of labeling IEG promoters.

#### 4.8.1 E-SARE

The enhanced synaptic activity-responsive element (E-SARE) consists of five tandem repeats of the SARE enhancer, which was previously discovered to be responsive to neuronal activity, fused to the minimal Arc promoter (Kawashima et al., 2013; Figure 2B). Compared to the Fos promoter, E-SARE demonstrates 30-fold higher reporter expression and 20-fold higher dynamic range in cultured cortical neurons. Moreover, the use of E-SARE increased the number of activated neurons by 8-fold *in vivo* (Kawashima et al., 2013). To label neural circuits activated during behavioral stimuli in a permanent manner, the E-SARE promoter was used to drive the expression of TM-inducible Cre recombinase, which was combined with the expression of a floxed fluorescent reporter.

#### 4.8.2 RAM

The robust activity marker (RAM) is a synthetic promoter consisting of four tandem repeats of synthetic enhancer modules (EMs) located upstream of the human minimal Fos promoter (Figure 2A). Each EM comprises the activator protein 1 (AP-1) binding site along with the binding motif from the *Npas4* gene, which is known to be enriched in DNA activity-regulated enhancers in the brain. These EMs were integrated into transcriptional regulatory sequences with a favorable secondary structure for transcription activation (Sørensen et al., 2016). The RAM promoter (PRAM) displayed an ~3-fold higher induction ratio in neuronal culture compared to the E-SARE promoter because of the higher basal activity of E-SARE (Sørensen et al., 2016).

In 2016, Sørensen et al. developed the RAM system with Cre recombinase (CRAM) to label specific GABAergic neurons in mice (Sørensen et al., 2016). It is a synthetic neuronal activity-dependent promoter with very low expression in basal conditions before a designated experience and is strongly induced by neural activity during the experience for robust ensemble labeling (Sørensen et al., 2016). Moreover, to identify Fos- and Npas4-dependent ensembles within the DG contextual fear memory engram, Sun et al. used their RAM reporter system to create Fos-dependent RAM (F-RAM) and Npas4-dependent RAM (N-RAM) reporters (Sun et al., 2020).

### 4.9 Summary of chemical-based activity-dependent tools based on immediate early genes

As fundamental tools to study neuronal ensembles involved in memory engrams, IEG-based systems can serve as a reliable marker for neuronal activation under a stimulus. In contrast to traditional genetic strategies, the expression of different IEGs involves distinct signaling pathways and therefore differs among neuron types and regions. This diversity enables the study of pairwise connections between brain regions (Nagel et al., 2003; Liu et al., 2012; Giannotti et al., 2019). The temporal resolution of chemical-based systems is constrained by drug delivery methods and pharmacokinetics in the brain (Barykina et al., 2022). For instance, the transcription of IEG proteins in the activated neurons is temporally limited. The expression of commonly used IEGs, such as Fos, Egr-1, Arc, and Npas4, peaks within 30 min and subsequently declines within 120–240 min following stimulation, depending on the specific stimulus (Greenberg et al., 1986; Guenthner et al., 2013). The mRNAs transcribed from IEGs exhibit a brief half-life, with Fos, for example, lasting ~10–15 min (Sheng and Greenberg, 1990). Other limitations include the non-universal nature of IEG response; IEG is expressed differently across different types of neurons in the brain. Guenthner et al. reported the recombination frequency was higher in ArcTRAP mice than in FosTRAP mice in most brain regions with the TM treatment; however, FosTRAP was more efficient in the cerebellum and thalamus (Guenthner et al., 2013). Viruses carrying activity-dependent tools based on IEGs are considered ideal vectors in diverse experimental animals. However, the effectiveness of infection is still insufficient, and several genetic strategies carried out by viruses are complex and cannot be revealed easily. Thus, developing more transgenic mice could solve these problems.

## 5 Activity-dependent tools for longitudinal records of cellular events

### 5.1 Background

Although several existing activity-dependent tools can tag activated engram neurons, they can only provide a snapshot of current neuron states during a user-defined time window (Barykina et al., 2022). Hence, there exists a need to create a tool that can longitudinally record the dynamics of neural events to study different physiological mechanisms of spatial and temporal patterning, such as the formation of engram neurons. To solve this problem, certain scientists described two cellular events recording systems based on self-assembling protein filaments (Lin et al., 2023; Linghu et al., 2023).

### 5.2 Principle

**Figure 3:**
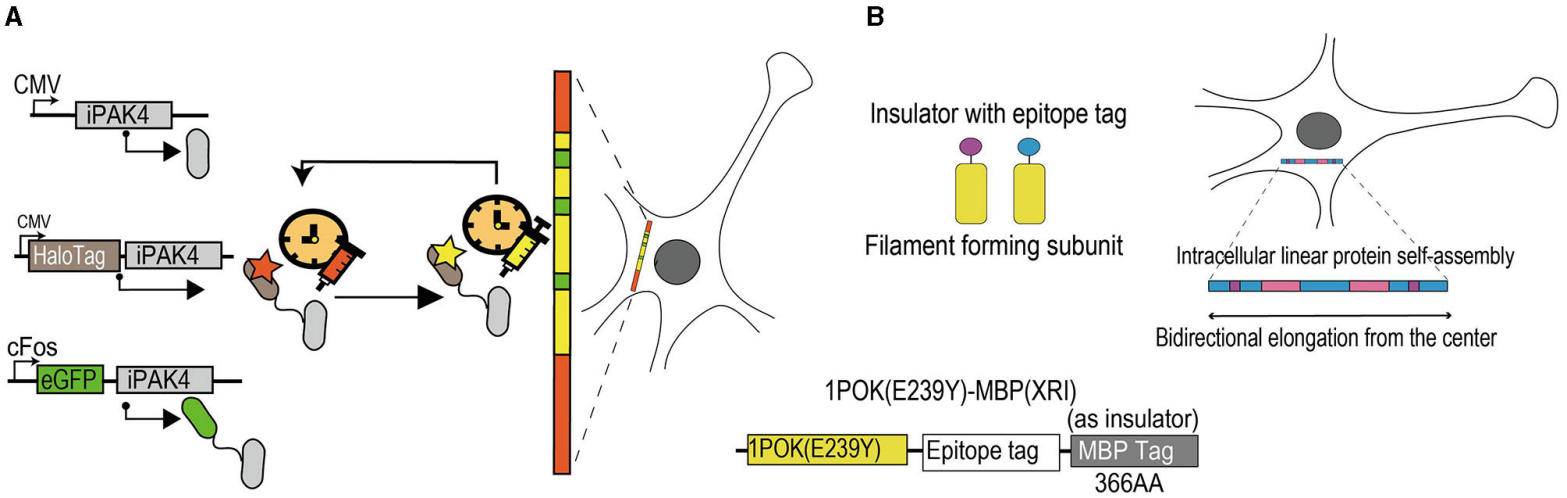
Schematic diagram of activity-dependent tools for longitudinal records of cellular events. **(A)** Experimental diagram of iPAK4-based systems to record cFos-driven transcription events. IPAK4, as a protein scaffold, can safely grow in mammalian cells over time. The HaloTag-iPAK4 can stably bind to the HT dye and achieve fiducial timestamps to label fibers without affecting the physiological stage of the cell. The cFos promoter can drive the expression of EGFP-iPAK4. The red and yellow areas on the protein filament mean fiducial timestamps and the green areas mean records of cFos-driven transcription events. IPAK4, a fusion of the catalytic domain of the Pak4 kinase and the 38 amino acid iBox domain of its inhibitor Inka1. EGFP, enhanced green fluorescent protein. CMV, cytomegalovirus. HaloTag, a protein tag that can combine with multiple fluorescent dyes. **(B)** Schematic diagram of XRI strategy. The XRI is a genetically encoded self-assembling protein system and mainly consists of three parts. The 1POK (E239Y), as a filament-forming subunit, plays a role in self-assembling, and the MBP tag effects in blocking the unwanted lateral binding and growth of the protein assembly as an insulator. The epitope tag connects the two parts together. When cells are alive, self-assembling tagged proteins are continuously added to the growing chain, allowing for continuous recording of the presence of different tagged proteins available carrying specific promoter and reporter genes. In 2 weeks, the result of the growing chain can be obtained through immunofluorescence imaging. The blue areas on the growing protein chain represent components on the self-assembled protein that change expression over time, as the background. The pink and purple areas represent components of the self-assembled protein that is driven by cellular events of interest. 1POK (E239Y), self-assembling protein variant. MBP, maltose-binding protein.

Lin et al. (2023) developed a protein-based recording system consisting of three components (Figure 3A). The first component is iPAK4, which is a protein scaffold that can safely grow over time in mammalian cells and combine with fluorescent labels without affecting the physiological stages of cells. The second refers to a method to add baseline timestamps to combine scaffold growth with the timing of events according to the external environment and correct for inevitable variations in the scaffold growth rate over time and between different cells. Third is a reporter gene of cellular events that can bind to the scaffold during a cellular event. The position of the activity-induced labels related to the fiducial timestamps is measured to infer the timing of the cellular events. It cannot be influenced by cell-to-cell variations. Before filaments approach whole-cell size, for about several days, this system can realize multi-day recordings of cellular events of interest with sub-hour temporal resolution.

The XRI strategy, a completely genetically encoded approach, consists of three components (Figure 3B). First is a human-designed filament-forming protein called 1POK (E239Y), which is selected following its expression in primary cultures of mouse hippocampal neurons. It can be induced to reliably generate continuously growing linear chains in mammalian cells with low immunofluorescence background. Second is a maltose-binding protein (MBP); it is an *Escherichia coli* protein that is usually used as a solubility tag for recombinant protein expression in animal cells (Kapust and Waugh, 1999; Reuten et al., 2016). Scientists have used the advantage of linear protein assembly, enabling reading out of useful information coding, and have fused the MBP to the lateral edge of the filament-forming monomer by epitope tag, which is the third component, and sterically blocked the unwanted lateral binding and growth of the protein assembly. To realize the longitudinal records of cellular events, scientists combined specific promoters and reporter genes with this module and transduced it to cultured neurons and experimental animals by virus. Any cellular events driven by transduced promoters are labeled by reporter genes within 2 weeks of virus transduction, and these results could be acquired by immunofluorescence imaging.

### 5.3 Selected examples of applications

Lin et al. (2023) designed the iPAK4 system and validated the longitudinal record of Dox-induced reporter gene expression in HEK cells and phorbol 12-myristate 13-acetate (PMA)-induced c-fos expression in cultured neurons. Both were transduced via CMVs. In addition, except for delayed transcription, translation, and protein folding, Lin et al. found that this system can infer an onset time for recorded cellular events at an average absolute timing accuracy of < 1 h. This tool has not been validated in experimental animals as it uses intracellular dyes to map fiducial timestamps.

Linghu et al. (2023) designed the XRI strategy and validated the XRI-record time courses of tamoxifen-induced gene expression and KCl-induced c-fos expression in cultured neurons. They (Linghu et al., 2023) found that the reporter gene expression recorded by XRI was positively related to KCl-stimulated concentration, as evident from time-lapse imaging. Moreover, XRI has been used in the living mammalian brain following transduction by AAVs and administration of 4-OHT after 10 days. The immunofluorescence imaging of the experimental brain slices after 14 days was similar to that observed in cultured neurons. The XRI strategy has verified its safety for cultured cells and experimental animals.

### 5.4 Summary of activity-dependent tools for longitudinal records of cellular events

Both systems are based on protein filaments that can grow and assemble intracellularly, continually add different marks to record cellular events, and read out temporal messages microscopically according to the locations of filaments. Differently, the XRI system is completely genetically encoded and has lower temporal resolution compared with the iPAK4-based system that uses exogenous dyes to incorporate into the filaments. The rapid growth rate of the filaments confers a lower time course to the iPAK4-based system than the XRI system (Burgess, 2023). Hence, future work involves improving the temporal resolution and prolonging the growth of filaments safely. In addition, the XRI system has been validated by tamoxifen-induced reporter gene expression both *in vitro* and *in vivo*, whereas the iPAK4-based system is only verified *in vitro*. Replacing the exogenous dyes with harmless components could broaden the application of the iPAK4-based system *in vivo* as well.

## 6 Discussion

Intrinsic cellular excitability, the propensity of a neuron to be activated by experience, plays a critical role in neuronal allocation. Furthermore, it is crucial to process that involves the recruitment into the engram (Guskjolen and Cembrowski, 2023). Based on a series of biochemical changes occurring in the activated neurons, scientists have designed several activity-dependent tools against different targets. For example, the CaM/CaMKII signaling pathway (extended data Figure 1). The activity-dependent tools based on calcium work on the principle that the activation of the neurons opens the voltage-sensing Ca^2+^ channels, NMDA receptors, and ryanodine receptors from the endoplasmic reticulum, triggering the intracellular Ca^2+^ concentration to increase and binding of Ca^2+^ to CaM. Thus, these tools can sense the change in the intracellular Ca^2+^ by calcium indicators and demonstrate that the neurons were activated by uncaging light-sensing molecules and photoswitching. The CREB-based activity-dependent tools, also called the CREB regulating systems, primarily focus on controlling the CREB activity to mediate the neuronal activity artificially. Activation of the neurons causes the phosphorylation of CREB by various kinases, such as CaMKII, and combined with specific sequences to induce IEG expression. The CREB is a necessary factor mediating the intrinsic neuronal excitability (Han et al., 2007), and overexpressing it would improve the possibility for neurons to become engrams. Hence, a combination of CREB regulating systems and other genetic strategies is indispensable for scientists to study engram neurons and memory formation. IEG-based activity-dependent tools, which are proxies of neuronal activity, are developed by combining the IEG promoters with other genetic systems, such as drug-induced systems. Due to their excellent robustness and construction using virus and transgenic mice, these tools are widely used to explore several difficult but meaningful neurobiological questions, especially in the general progression of memory and engram theory. Most activity-dependent tools can only provide a snapshot of dynamic processes of the formation of engram cells or the formation of memory at a specific time but cannot record and study their dynamic mechanisms over time. Scientists have developed two systems that can longitudinally record cellular events based on protein filaments, which can provide new powerful tools to study complicated scientific questions.

A crucial step in the study of engram cells is to have an exact definition and criteria combined with experimental results. The classical criteria proposed by Semon are summarized as four words, including persistence, ecphory, content, and dormancy (Josselyn et al., 2015). Persistence refers to continuous changes in engram cells activated by experience. Based on that, scientists developed activity-dependent tools to label the activated neurons and manipulate their activity by genetic strategies, including optogenetics and DREADDs, thus successfully validating the criteria of ecphory and content. The dormancy of engram cells was achieved by Liu et al. (2012), who used optogenetics to reactivate the hippocampal engrams and successfully recalled the fear memory of mice, which failed to retrieve by natural cues. To improve the temporal–spatial resolution and manipulate engram cells more accurately, activity-dependent tools were developed and combined with drug-induced systems and light-gated systems. The use of light-gated components provides activity-dependent tools based on calcium with an ideal time window of minutes (Barykina et al., 2022). Furthermore, scientists have found numerous new conceptions of engram cells and limitations of activity-dependent tools. First, the multiple trace theory states that in addition to activated cells during the user-defined time windows, non-activated cells would become the engram cells during memory consolidation and retrieval (Nadel et al., 2000). The actively inhibited cells involved in other processes of memory (Nomura et al., 2015; Vetere et al., 2021) should be appreciated during the formation of engram cells. Thus, labeling and manipulating inactivated cells around the activated cells is crucial for studying memory engrams. However, the available activity-dependent tools can only tag activated cells. The longitudinal record systems of cellular events by protein filaments can be used to resolve this problem. In addition, identifying an activity-dependent proxy of actively inhibited neurons will assist in the development of activity-dependent tools. Second, astrocytes, as non-neural cells, are prime candidates to become engram cells. Astrocytes have been implicated in memory allocation and retrieval (Adamsky et al., 2018), suggesting the involvement of neuroglia in engram allocation and the potential to become engram cells. Thus, researchers should also focus on neuroglia to develop additional activity-dependent tools. Third, certain engram cells can only participate in one stage of memory. Studies have demonstrated that certain neurons function in memory encoding or retrieval but do not play a similar role in another stage (Roy et al., 2017; Cembrowski et al., 2018; Quinones-Laracuente et al., 2021; Lee J. et al., 2023). This finding supplements the criteria of engram cells proposed by Semon that certain engram cells display ecphory, whereas others could be involved in one process of memory. Although the currently available activity-dependent tools can label and manipulate that kind of engram cells, most scientists studying the engram cells focus on reactivated engram cells during memory retrieval. The longitudinal record of cellular events can be used to answer interesting questions, such as those on the formation of different engram cells.

Transcriptome analysis has been widely used in biological research. To explore the sustained chemical and physical changes in engram cells (Rao-Ruiz et al., 2019), combined with Arc-reporter gene transgenic mice, scientists have performed unbiased RNA sequencing of DG engram neurons 24 h after conditioning and identified CREB-dependent transcription features between these neurons during memory consolidation. Thus, the future development of activity-dependent tools involves not only improving the characteristics of their own components (Lee S. et al., 2023) but also combining them with other useful molecular technological methods (Guzowski and Worley, 2001; Wang et al., 2009; Li et al., 2010; Guez-Barber et al., 2011; Chung et al., 2013; Riedy and Keefe, 2013; Hachem-Delaunay et al., 2015; He et al., 2019; Vassilev et al., 2020). Only tagging the engram cells is not sufficient; it is also essential to correctly analyze them at the behavioral diagrams and molecular levels to promote our understanding of engram cells and memory.

## Author contributions

BP: Conceptualization, Software, Investigation, Writing – original draft, Writing – review & editing. XW: Conceptualization, Software, Investigation, Writing – original draft, Writing – review & editing. HC: Conceptualization, Software, Investigation, Writing – original draft, Writing – review & editing. YY: Conceptualization, Software, Resources, Investigation, Writing – original draft, Writing – review & editing. ZD: Resources, Software, Investigation, Writing – review & editing. ZY: Resources, Software, Investigation, Writing – review & editing. XY: Conceptualization, Writing – review & editing WW: Conceptualization, Funding acquisition, Writing – review & editing. KL: Conceptualization, Funding acquisition, Writing – review & editing. All authors read and approved the final manuscript.

## 7 Glossary

AD, Alzheimer's disease; IEGs, immediate early genes; CREB, cAMP response element binding protein; GECIs, genetically encoded calcium indicators; GPCRs, G protein-coupled receptors; LOV, light-oxygen-voltage; eLOV, evolved light-oxygen-voltage; hLOV1, hybrid LOV domain; CaM, Calcium-sensing calmodulin; tTA, tetracycline-controlled transactivator; rtTA, reverse tetracycline-controlled transactivator; rsCaMPARI, Reversibly Switchable CaMPARI; UV, Ultra Violet; TEVp, tobacco etch virus protease; TEV, C-C-terminus of split TEVp; TEV, N-N-terminus of split TEVp; TEVcs, TEV cleavage site; ST-Cal-Light, Soma-Targeted version of Cal-Light; FLiCRE, Fast Light and Calcium-Regulated Expression; scFLARE, Single-chain FLARE; CaTEV, calcium-dependent TEV protease; DG, dentate gyrus; LA, lateral amygdala; TetTag, Tetracycline Tag system; CPP, conditional placed preference; PL, prefrontal cortex; TM, tamoxifen; CN, central nucleus of the amygdala; CANE, capturing activated neural ensembles; dsTVA, destabilized TVA; RV, rabies virus; LV, lentivirus; vGATE, virus-delivered Genetic Activity-induced Tagging of cell Ensembles; E-SARE, The Enhanced Synaptic Activity-Responsive Element; RAM, The Robust Activity Marker; EMs, enhancer modules; AP-1, Activator Protein 1; PRAM, The RAM Promoter; CRAM, RAM system with Cre recombinase; F-RAM, Fos-dependent RAM; N-RAM, Npas4-dependent RAM; MBP, maltose binding protein; PMA, phorbol 12-myristate 13-acetate; OT, optogenetics; NAc, nucleus accumbens; VTA, ventral tegmental area; vCA1, ventral hippocampus CA1 regions; TRAP, Targeted recombination in active populations; 4-OHT, 4-hydroxytamoxifen; BAC, bacterial artificial chromosome; Dox, doxycycline; CMV, cytomegalovirus; CaMPARI, Calcium-modulated photoactivatable ratiometric integrator.
