# Supplementary figures and images for: Exploring the memory: existing activity-dependent tools to tag and manipulate engram cells
Source: Front Cell Neurosci. 2024 Jan 8;17:1279032. doi: 10.3389/fncel.2023.1279032 (PMC10800721; doi:10.3389/fncel.2023.1279032)

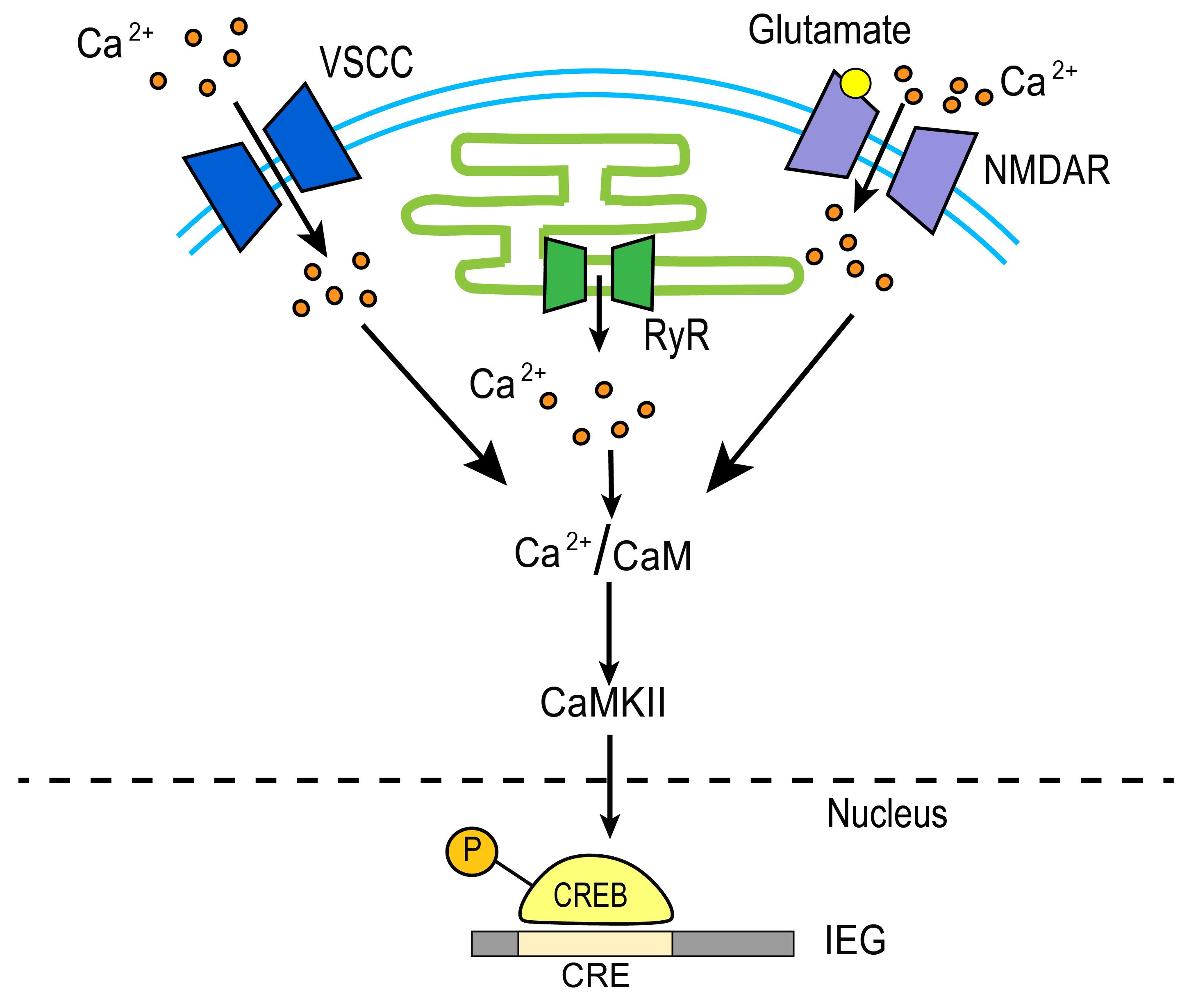

Supplement: Supplementary file 1 [file Image_1.jpg]
